# Understanding the process of social network evolution: Online-offline integrated analysis of social tie formation

**DOI:** 10.1371/journal.pone.0177729

**Published:** 2017-05-24

**Authors:** Doyeon Kwak, Wonjoon Kim

**Affiliations:** 1 Graduate School of Culture Technology, KAIST, Daejeon, Republic of Korea; 2 School of Business and Technology Management, KAIST, Daejeon, Republic of Korea; Universidad de Zaragoza, SPAIN

## Abstract

It is important to consider the interweaving nature of online and offline social networks when we examine social network evolution. However, it is difficult to find any research that examines the process of social tie formation from an integrated perspective. In our study, we quantitatively measure offline interactions and examine the corresponding evolution of online social network in order to understand the significance of interrelationship between online and offline social factors in generating social ties. We analyze the radio signal strength indicator sensor data from a series of social events to understand offline interactions among the participants and measure the structural attributes of their existing online Facebook social networks. By monitoring the changes in their online social networks before and after offline interactions in a series of social events, we verify that the ability to develop an offline interaction into an online friendship is tied to the number of social connections that participants previously had, while the presence of shared mutual friends between a pair of participants disrupts potential new connections within the pre-designed offline social events. Thus, while our integrative approach enables us to confirm the theory of preferential attachment in the process of network formation, the common neighbor theory is not supported. Our dual-dimensional network analysis allows us to observe the actual process of social network evolution rather than to make predictions based on the assumption of self-organizing networks.

## Introduction

In an age of perpetual digital connectedness, our ways of bonding and maintaining relationships heavily depend on online social networking. More than one billion users around the world are actively networking through Facebook, one of the most prominent social networking services, to promote their online social existence and connections [1]. Social networking services (SNS) provide powerful tools for generating social capital, as they allow users to develop new connections and expand their personal networks [2]. Here, possessing a durable social network can provide network participants with otherwise unattainable resources, such as access to information, financial gains and psychological well-being [3,4,5]. As social networks can provide direct or indirect benefits for network participants, understanding how new social ties form to expand networks has been an important research topic.

To understand the formation of new ties within a network, various researchers have attempted to visualize the evolution of a network by utilizing quantitative social network data available on popular social networking platforms [4,6]. By understanding the structure of a network and the characteristics of its participants available on SNS, researchers have been able to predict the probability of new link formations via preferential attachment model and common neighbor analysis. The preferential attachment model by Albert and Barabasi explains that the number of connections a network participant has, or the degree centrality that a node has, can act as a strong indicator for new network connections to the node [7]. Additionally, common neighbor analysis suggests that having a common neighbor or a mutual friend between a pair of interacting network participants can facilitate the connection between the pair [8]. These prediction models utilize quantitative factors from initial network structure based on SNS to predict new link formations within a network.

However, previous studies have only devoted limited attention to exploring the evolution of a network from the online network perspective, although most networks develop through interaction among network participants both online and offline. In other words, existing studies portray the concept of social network ties as online representations on SNS of various communications among network participants over series of time, which is also based on traditional offline interactions. Thus, the limitation of previous studies is that the formation of social network may be associated with off-grid interactions among individuals apart from interactions revealed strictly in an online social network. In particular, network connections on Facebook are generally known to form among offline acquaintances rather than through online interactions, as the use of SNS to keep in touch with people whom they already knew tended to outweigh the use of SNS to meet new people [9]. Although the traditional models explain how networks can self-organize according to prior characteristics through preferential attachment model and common neighbor effects, these social network analysis techniques predict social network evolution from a one-dimensional online network perspective [6].

Therefore, to understand the significance of interrelationships between online and offline social factors in generating social ties, we analyze the associated online and offline components in the process of making new online social links. Analyzing the offline interactions among network participants and monitoring the changes in their online social networks allow us to re-confirm how online factors can predict the generation of new social ties as well as to visualize how offline interactions influence the evolution of social network. Both online and offline dimensions of our social network are the fundamental grounds for interacting, maintaining, and recording our social relationships. Hence, it is important to consider the two-dimensional nature of our social network when understanding the formation of new ties and the process of social network evolution.

Acquiring quantitative data available on online network has become much easier due to SNS, and thus, recent prediction models have utilized online data. However, in consideration of the interweaving nature of online and offline networks in generating social capital in reality, quantifying social factors from the offline domain is just as crucial to understanding how social interaction accumulates and transcends across the on-offline environment [10]. Agarwal, Gupta and Kraut further argues that technologically mediated interactions cannot replicate the physical and emotional presence of offline interaction [11], and the online social network is a representation of the preexisting offline network [5,12,13]. Thus, we believe that offline interactions stimulate the growth of online interactions, and it is necessary to quantify the unmeasured offline interactions that are involved in the evolution of an online social network.

Thus, to observe the actual process of building new links, we study not only factors from existing online network structures but also offline interactions that promote social linkages. In this study, we analyze the offline interaction data from a series of 7 monthly wine parties (offline social events) which measured the interaction between the participants using radio signal sensors. We also collected information on online network factors before and after the events to reveal the process of social network evolution. By observing the link formation process through both aspects of our social networks, we verify the effects of prominent prediction models: the preferential attachment model explaining new link formations via the degree of participants and the common neighbor effect that explains link formations via mutual friendships.

## Theoretical background

### Dual-dimensional online and offline social network model

**Fig 1:**
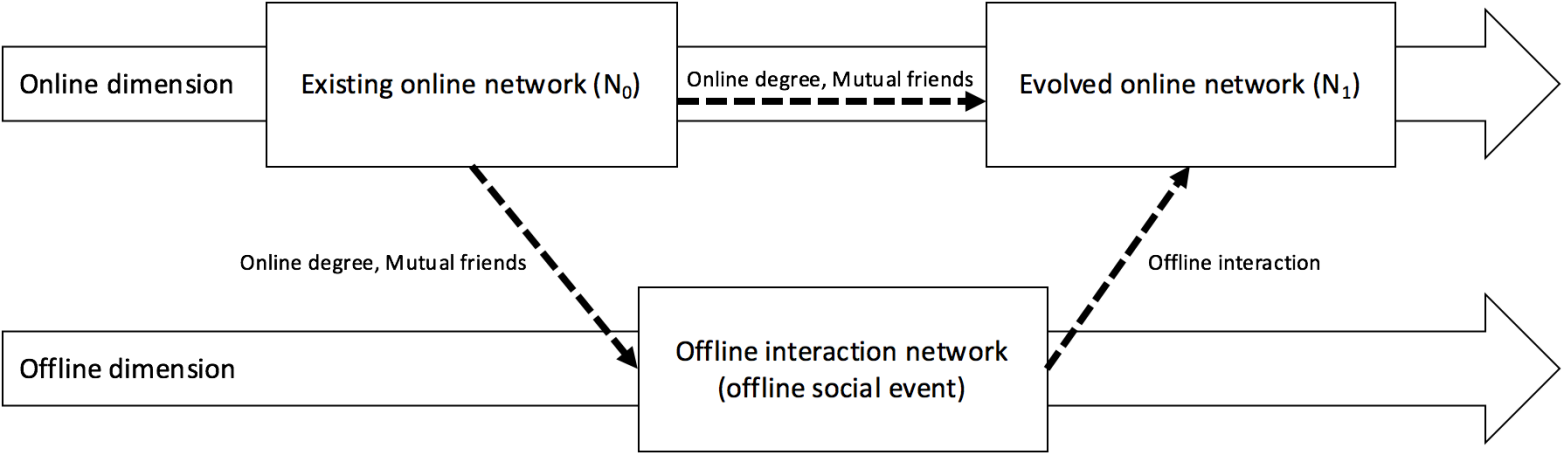
Evolution of an online network with a known instance of an offline social event. Online network link formation can be predicted using prior online factors such as degree and the presence of mutual friends. These factors also affect offline social interaction at a social event, which in turn will directly affect the online link formation.

In our research, we examine a multilayered online-offline network [14,15] to confirm the effect of online predictors with offline social interaction (Fig 1). As the embedded resources in a network can be accessed for social benefits [16], we examine how the social structural resources from existing online networks affect interaction behaviors at offline events and the final resulting online social network. These embedded resources within a network structure are measured through degree centrality [17], which captures the effects of preferential attachment, and mutual friendships between a pair of social participants, which capture the effects of common neighbors. The number of online connections among individuals, which measures their degree centralities and presence of mutual friends on Facebook between the pair at a social event, acting as a bridge between the two individuals, may affect the connection between the pair [8].

### Preferential attachment model

Previous studies have suggested that the more connected a network node is or the greater degree centrality a node has, the more likely it is to receive new connections [18]. This theory, i.e., the preferential attachment model, is one of the most prominent models that incorporate the growth of a network and preferential or biased network connections based on a node’s attributes. If a social network participant were to connect with another participant randomly, the probability of connecting with a participant would be proportional to his/her degree [19]. The preferential attachment explains a positive feedback loop where initial differences in degree centrality are automatically reinforced, eventually magnifying the differences.

Although there have been ample discussions of preferential attachment at the level of online social networks [18,20,21], the underlying mechanism explaining how this model develops at the offline level has not been well-explored. It is expected that the offline interaction between participants will significantly affect the formation of a new online social tie between them, and that it is less likely for online social ties to form without this offline interaction. In other words, we expect that the evolution of online social networks significantly depends on the offline interaction that is not reflected in the online network layer. Many studies indicate that online networking sites are used to maintain and reinforce offline relationships, as opposed to establishing new relationships online [5,22]. There is a tendency for individuals to group when interpersonal relationship are positive, which indicates that frequent positive interactions lead to the formation of a network [23,24]. Furthermore, Sykes’ research suggests that previous acquaintances have a strong effect on the frequency of interaction, demonstrating that prior social connections can influence new connections [25]. Offline interaction may be a significant underlying mechanism of preferential attachment, which we empirically explore through randomized controlled experiments of offline social events.

### Effect of common neighbors

In addition to preferential attachment, people tend to make social connections by introducing pairs of their friends to one another, thus completing triangles in a network and increasing the clustering coefficient [26]. Newman constructed a growth model of a social network in which one preferentially makes links between pairs of individuals who have one or more common neighbor, or mutual friends. The network shows clear clusters of nodes that share many connections internally, and fewer shared outside the group. The common neighbor analysis reflects a mechanism for local community formation in social networks in which network participants introducing pairs of their friends to one another induce cliquishness and social groupings.

This common neighbor theory also significantly explains the evolution of social networks but explores the issue from a one-dimensional network perspective [21]. By incorporating offline social interaction, we can understand the process of how common neighbor effects drive the formation of social ties between once-unknown participants.

We believe that the presence of mutual friends is crucial in terms of introducing and bringing together participants who are not yet connected. However, in a social setting where all participants have chance to meet and interact with one another regardless of having common acquaintances, the effect of common neighbors can provide contradictory results. On the one hand, the presence of mutual friends may positively affect the interaction between participants not yet connected, as mutual friends can mediate and induce interaction between them. On the other hand, if the role of mutual friends is solely providing a chance for unlinked strangers to meet each other, then the presence of mutual friends may have no significant effect in an offline social setting where all participants have a chance to meet one another on their own. Moreover, the presence of mutual friends may provide distraction for the pair of interacting participants [27,28]. Mutual friends may indirectly hinder the interaction between participants who are not yet linked by competing for interaction time within the limited duration of our social events.

Introducing intermediary offline social interactions into the network evolution will illuminate the underlying mechanisms for these online social predictors. By taking offline social behavior into account, we can verify and explain the process of these online predictors affecting online social tie formation in a multilayered on-offline social network. We will conduct a regression analysis with new tie formation between a pair of interacting social participants as the dependent variable to understand how online predictors and offline interaction affect network evolution.

## Methodology

### Data structure

**Fig 2:**
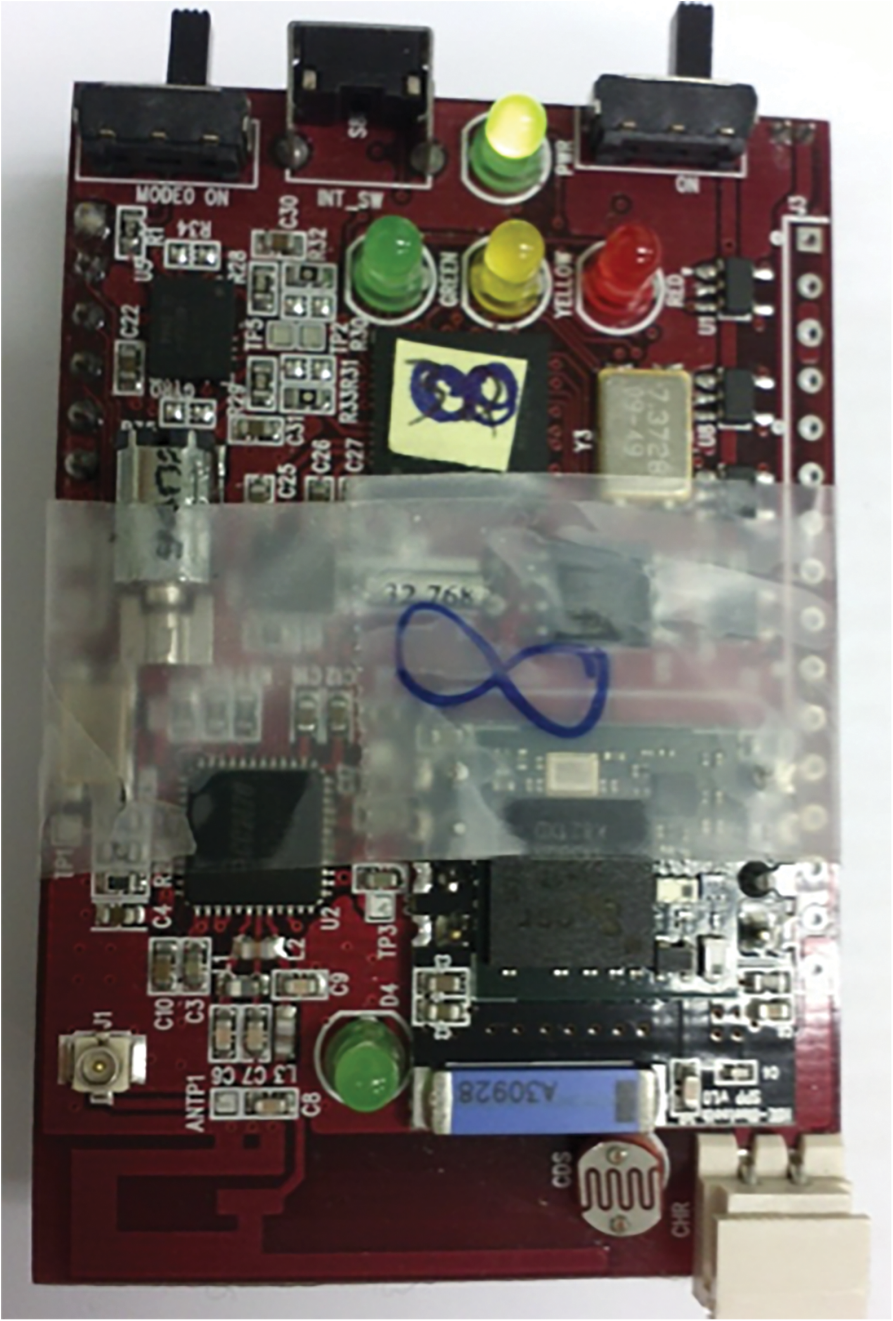
Portable radio signal strength indicator device used to measure radio signal strength from other devices. Social event participants wore necklaces carrying the devices.

Our research is primarily based on the 7 monthly social networking events hosted by AtDusk, a social event organizing company, from November 2011 to May 2012 on the first Fridays of the months. AtDusk promoted the social events through Facebook advertisements in both Korean and English. Each event lasted approximately 3 hours and had approximately 30 participants in attendance, including many local business owners, professionals, and undergraduate and graduate students in Daejeon, Korea (Table 1). The series of social events consisted of participants each carrying a radio signal strength indicator device (RSSI), compatible with IEEE 802.15.4 Protocol (Zigbee), which detects the strength of radio signals from other participants’ devices to measure the proximity and duration of interactions (Fig 2). The RSSI devices were used to detect social proximity because they can cover almost 180 degrees of forward-facing direction to detect all interacting social participants, including those talking side by side. Moreover, radio signal strength decreases when blocked by a human body, which we used to our advantage, to detect only forward-facing interactions when sensors were worn as necklaces. In this way, the radio signal strength indication can show stronger signal transmission between conversation members than with non-members at the same distance.

**Table 1:** Descriptive statistics.

	Individual level	Pair level	
**Attendance**			
Total	230 (123 unique)	3703 pairs	
Party 1	34	561	
Party 2	36	630	
Party 3	27	351	
Party 4	37	666	
Party 5	35	595	
Party 6	30	435	
Party 7	31	465	
**Gender**			
Female (F)	82	FF	413
Male (M)	148	MF	1662
		MM	1628
**Language**			
Korean (K)	95	KK	648
Bilingual (I)	32	KE	1409
English (E)	103	KI	400
		II	56
		IE	450
		EE	740

Descriptive statistics of event participants. The data used for this research is pair-level data which consists of all instances of two individuals who have interacted within a social event. This data is all detected interacted pairs, not all possible pair combinations.

Four transmission receiving stations were installed in each corner of the event location to control the interaction proximity. The measured values were partitioned into various conversational groups using the K-means clustering method. The *k* centroids were selected among the nodes of individual attendees, and because the number of groups (*k*) had to be predetermined, *k* was updated proportionally to the total number of attendees at the event. We were able to detect which two or more nodes were within the same cluster, signifying the social interaction among individuals within the same groups. When compared to the ground truth results from the manual annotation of two months of event video recordings with no blind angles, our data showed a random index value of 0.872 [29], which indicates that the sensor-based method demonstrates high accuracy in measuring offline interaction for our research purposes.

We have not only acquired the participants’ offline social interaction measurements from the 7 social networking events, as mentioned above, but also followed the changes in their online networks using a crawler scripted in Sikuli, a Python-based compiler that uses images to recognize graphical user interface, to crawl publicly available personal and friendship information on Facebook [30]. Although Facebook provides publicly available demographic data of all users through their Facebook API, they do not provide information on the date that the friendship was made. Our Sikuli script generates URLs of Facebook friendship pages between all possible pairs of participants who have attended the social events using their Facebook ID. The crawler goes through all possible friendship pages to detect pairs’ friendships and dates of friendships by recognizing and recording the names and dates of friendships when available. The names of the participants, Facebook IDs and the generated URLs are automatically deleted after the data collection, and undisclosed to the researchers to protect participants’ privacy. With the collected data, we modeled existing online networks prior to each event and online networks after each event. Within the bounds of the Facebook privacy settings of the participants, we were able to collect online samples from 244 unique participants with 3703 possible interaction pair occurrences (unbalanced panel data) throughout 7 social events.

### Privacy considerations

All of the social event participants were informed in advance by AtDusk, the social event organizing company, through promotional materials, written informational document and oral explanation in Korean or English that interaction sensing technology would be deployed at the event and their publicly available Facebook data would be gathered, to provide event participants their personalized interaction statistics via AtDusk Partylytics Facebook application [31]. The participants were able to enter the social events after they have given the verbal consent and registered into Partylytics, using their Facebook ID and password. In June 2016, this interaction data was disclosed to us, researchers for researching purposes with the consent from Atdusk and the event participants at the selected social events. Thus, the given data was purely an observational data, collected on the real social event settings organized by the company, AtDusk. Therefore, there were no instructions for the participants to perform specific tasks regarding the experiment, and the company has only monitored real interaction behavior in real life situations. Thus, the social event participants were not experiment participants, as they came to the events to enjoy themselves [29]. Their relevant demographic information, such as gender, spoken language, and friendship ties only between the social event participants, was collected through AtDusk and publicly available data on Facebook, which does not violate participants’ privacy. The names, Facebook IDs of the participants, and the generated URLs, although publicly available on Facebook, were automatically deleted through our scripted crawler after recognizing their social ties on Facebook. All of the data used in this research complies with the terms of services of Facebook and AtDusk. We did not have any access to the participants’ private information which may allow us to verify individuals who have participated in the series of social events.

### Variables

#### Dependent variables

Our dependent variable is the newly made social linkage, *Made Friends*, of the 3703 pairs. This binary variable is measured via Facebook friendship data. If a friendship is created within one month after the date of an event between a pair who participated, the value is recorded as 1; otherwise, it is 0. There were 240 occurrences of *Made Friends* between 3703 possible pairs over 7 social events (Table 1).

#### Independent variables

Our independent variables are the online *Degree* of a pair of event participants in the online social network prior to the social event, the presence of online *Mutual Friends* shared by a pair, and the offline *Interaction* between a pair of nodes in an offline social event. These variables best represent major factors of online and offline social networks. The *Degree* variable between a pair is calculated by measuring the combined degree centralities of 2 nodes of the pair while subtracting the number of mutual friends shared by the pair which is counted twice, and excluding the connection between the pair if it is present. The *Degree* of a pair measures all participants connected to the pair through the online social network prior to the social event. Among our studied 3703 pairs over 7 social events, there are 12.59 participants on average linked to a given pair at a social event.

The presence of online mutual friends at an attended social event, *Mutual Friends* at the social event, is a binary variable. When at least one mutual Facebook friend is present at an event between a pair of participants, this value is 1; otherwise, it is 0. Because of an excess of 0 mutual friends shared by observed pairs, normalization of the distribution was not possible, and thus, the value was converted to a binary variable. There were 1502 cases of the presence of at least one mutual friend between 3703 pairs.

The *Interaction* variable between pairs was calculated utilizing data from RSSI sensors as mentioned above. This variable measures the total duration of interaction between a pair of participants. The interaction duration was measured every 10 seconds to prevent ‘pass-bys’ without actual interaction affecting our data. We also used log scale to prevent skewed distribution, and the *Interaction* variable was 90.78 (approximately 908 seconds) on average and in log scale, 1.751 for 3703 possible pairs.

#### Control variables

Since the *Made Friends* variable cannot be 1 when there is an already existing friendship between a pair, the *Already Friends* binary variable was used to control the regression outcome. The *Already Friends* variable between a pair of participants is 1 when a Facebook friendship has been made prior to the pair’s event attendance; otherwise, it is 0. A total of 805 cases of friendship existing prior to social events were observed in 3703 pairs.

As there were 7 consecutive but separate social events with some overlapping attendees and some new participants, the *Party ID* dummy variable was used to normalize our panel data. A total of 561, 630, 351, 666, 595, 435 and 465 cases of pair occurrences were observed. Demographic dummy variables *Gender* and *Language* were used to control the regression analyses. Gender was controlled as the interaction and Facebook friendship status may be affected by gender due to potential social status and romantic relationships. Language was controlled as the series of events were conducted in Daejeon, Korea targeting Korean and non-Korean international participants, and there may be possible effects of language barrier hindering participants from communicating with one another. *Gender* was categorized in 3 groups: female-female pair (FF), male-female pair (MF) and male-male pair (MM). A total of 413, 1662 and 1628 cases, respectively, were observed. *Language* used between a pair was categorized in 6 groups: Korean-Korean pair (KK), Korean-English pair (KE), Korean-bilingual pair (KI), bilingual-bilingual pair (II), bilingual-English pair (IE) and English-English pair (EE). There were 648, 1409, 400, 56, 450 and 740 cases, respectively (Table 1).

### Analysis models

To test our hypotheses, we used panel logit regression model to understand the effects of predictors from prior online network structure and offline interactions on the formation of social ties in the resulting online network.

Our main regression analysis utilizes panel logit regression with *Made Friends* as the dependent variable.

$$L_{ij,t}=b_{0}+b_{1}\cdot k_{ij,t-1}+b_{2}\cdot x_{ij,t}+b_{3}\cdot y_{ij,t}+b_{4}\cdot z_{ij,t}+e_{ij,t}$$ (1)

*L*_*ij*,*t*_ is the *Made Friends* binary variable between a pair *i* and *j* at social event *t*. *B*_*0*_ is the intercept estimate of the model. *B*_*1*_ is the coefficient of the *Degree* variable, which measures the degree centrality of a pair *i* and *j* (*k*_*ij*,*t-1*_), measured prior to the social event *t*. *y*_*ij*,*t*_ is the variable for offline *Interaction*, and *B*_*2*_ is its coefficient. *B*_*3*_ is the coefficient of the binary *Mutual Friend* variable, *x*_*ij*,*t*_. *B*_*4*_ is the coefficient of all control variables, *Already Friends*, *Gender*, *Language* and *Party ID* (*z*_*ij*,*t*_). *E*_*ij*,*t*_ is the error term of the regression. The analysis results will explain the influence of online predictors and offline interaction on new social tie formation from the pair perspective.

Since there may be causal effects of prior online network structure on offline interaction, we also perform panel linear regression with offline *Interaction* as the dependent variable to further verify the influence of online factors on offline behaviors.

$$y_{ij,t}=a_{0}+a_{1}\cdot k_{ij,t-1}+a_{2}\cdot x+a_{3}\cdot q_{ij,t}+u_{ij,t}$$ (2)

*y*_*ij*,*t*_ is the *Interaction* between a pair *i* and *j* at social event *t*, as mentioned above. *a*_*0*_ is the intercept estimate of the model. *a*_*1*_ is the coefficient of *Degree* centrality of a pair *i* and *j* (*k*_*ij*,*t-1*_). *a*_*2*_ is the coefficient of *Mutual Friends* for the social event variable, *x*_*ij*,*t*_. *a*_*3*_ is the coefficient of the control variables, *Already Friends*, *Gender*, *Language* and *Party ID* (*q*_*ij*,*t*_). *v*_*ij*,*t*_ is the error term for this regression. Based on the influence of online factors on the offline interaction, we can further investigate the reasons for our results from the primary regression.

## Results

### Correlation analysis

According to the correlation coefficients, there is no significant correlation problem. There are signs of slight collinearity between online *Degree*, *Mutual Friends* at the social event, and *Already Friends* variables (Table 2). This occurs, because the pure chance of having mutual friends and a pair already being friends at a given social event increase as the number of connections to Facebook nodes is more readily available, which accounts for the *Degree* variable.

**Table 2:** Correlations of variables (n = 3703).

	Variables	1	2	3	4
**1**	Friends Made^a^	-	-	-	-
**2**	Interaction, log(*y*_*ij*_ + 1)^b^	0.12**	-	-	-
**3**	Degree of a Pair	0.10**	0.08**	-	-
**4**	Mutual Friends^a^	-0.03	0.05**	0.46**	-
**5**	Already Friends^a^	-0.14**	0.16**	0.49**	0.42**

^a^ Binary variables composed of 0 or 1.

^b^ Log-treated variables have +1 within the logarithm function to prevent infinity.

** p<0.01

### Panel logit regression analysis (DV: Made Friends)

The panel logit regression with the dependent binary variable *Made Friends* tests the effects of offline *Interaction* and two factors from the prior online network: *Degree* and *Mutual Friends* (Table 3). *Already Friends* is the control variable for existing friendships prior to our events, and the *Party ID* control variable provides the fixed effect for independent social events. In this analysis, 7 models were tested; Models 1, 2 and 3 observe the correlation of the offline *Interaction*, online *Degree*, and *Mutual Friends* variables, while Models 4, 5 and 6 regressions test different combinations of two of the three variables. Model 7 observes all three independent variables. According to Model 1, the independent variable, offline *Interaction*, showed a significant result of 0.697 at p<0.01. This result confirms that a higher amount of offline interaction results in a greater likelihood of making a new social tie between two participants. Model 2 shows that there is a positive and significant estimated coefficient between *Made Friends* and the online *Degree* of a pair at 0.131 (p<0.01), which means that a pair of individuals having a greater number of connections has a greater chance of making a new connection with each other. However, Model 3 does not show the effects of the presence of *Mutual Friends* at a social event by itself, as it is nonsignificant, which does not confirm the theory of common neighbors promoting friendship. Model 6, which analyzes the prominent predictors of social network evolution without the offline *Interaction* variable, shows that *Degree* is positively associated with *Made Friends* at 0.139 (p<0.01), and *Mutual Friends* is negatively associated with *Made Friends* at -0.455 (p<0.01). Furthermore, when we observe Model 7 with the addition of the offline *Interaction* variable, we confirm once again the above findings. The *Interaction* coefficient is 0.798 (p<0.01), the *Degree* coefficient is 0.148 (p<0.01), and the *Mutual Friends* coefficient is -0.514 (p<0.01). Model 7 has the highest log-likelihood, indicating the best fit of the 7 models. This result verifies that the online degree centralities of the prior network can be used to predict social network formation while disproving the readily accepted notion of common neighbors within a network structure having a positive influence on new social link formation.

**Table 3:** Panel logit regression (DV: Made Friends).

Variables	Model 1	Model 2	Model 3	Model 4	Model 5	Model 6	Model 7
Intercept	-6.947***	-5.669***	-4.186***	-8.920***	-6.974***	-5.512***	-8.717***
(s.e.)	(0.738)	(0.646)	(0.624)	(0.800)	(0.747)	(0.647)	(0.799)
**Independent variables**							
Interaction log(*y*_*ij*_ +1)	0.697***			0.795***	0.697***		0.798***
	(0.092)			(0.101)	(0.092)		(0.101)
Degree		0.131***		0.138***		0.139***	0.148***
		(0.013)		(0.014)		(0.013)	(0.014)
Mutual Friend (binary)			0.044		0.041	-0.455***	-0.514***
			(0.157)		(0.164)	(0.167)	(0.175)
**Control Variables**							
Already friends (binary)	-17.733	-18.362	-17.583	-18.586	-1.752	-18.204	-18.419
	(613.191)	(575.294)	(638.904)	(555.760)	(613.241)	(568.037)	(547.871)
Gender MF	2.036***	2.024***	2.194***	1.810***	2.035***	2.025***	1.778***
	(0.593)	(0.594)	(0.592)	(0.597)	(0.593)	(0.594)	(0.596)
Gender MM	2.415***	2.434***	2.710***	2.0533***	2.412***	2.457***	2.044***
	(0.595)	(0.597)	(0.594)	(0.599)	(0.595)	(0.597)	(0.599)
Language IE	0.223	-0.131	0.014	-0.030	0.220	-0.132	-0.022
	(0.245)	(0.248)	(0.239)	(0.258)	(0.245)	(0.249)	(0.259)
Language II	0.735	0.224	0.444	0.452	0.733	0.226	0.461
	(0.493)	(0.505)	(0.479)	(0.520)	(0.493)	(0.508)	(0.524)
Language KE	-0.128	-0.293	-0.422**	0.013	-0.129	-0.284	0.033
	(0.193)	(0.193)	(0.187)	(0.199)	(0.193)	(0.194)	(0.201)
Language KI	0.603**	0.301	0.302	0.546**	0.604**	0.266	0.509**
	(0.242)	(0.243)	(0.233)	(0.252)	(0.242)	(0.245)	(0.255)
Language KK	-0.238	-0.034	-0.534**	0.255	-0.239	-0.011	0.307
	(0.264)	(0.270)	(0.258)	(0.275)	(0.264)	(0.271)	(0.277)
Party ID 2	-0.551**	-0.652***	-0.285	-0.895***	-0.544**	-0.746***	-1.000***
	(0.242)	(0.247)	(0.238)	(0.252)	(0.243)	(0.250)	(0.256)
Party ID 3	-0.686**	0.187	-0.174	-0.305	-0.676**	0.114	-0.416
	(0.284)	(0.283)	(0.274)	(0.295)	(0.287)	(0.285)	(0.299)
Party ID 4	-0.175	-0.413*	0.049	-0.610***	-0.161	-0.542**	-0.784***
	(0.213)	(0.222)	(0.212)	(0.228)	(0.219)	(0.227)	(0.237)
Party ID 5	-1.623***	-1.639***	-1.48***	-1.703***	-1.603***	-1.843***	-1.962***
	(0.321)	(0.321)	(0.320)	(0.327)	(0.330)	(0.331)	(0.340)
Party ID 6	-1.082***	-0.507*	-0.830***	-0.730***	-1.058***	-0.726**	-1.015***
	(0.273)	(0.274)	(0.279)	(0.281)	(0.289)	(0.286)	(0.297)
Party ID 7	-0.731***	-0.226	-0.440*	-0.447*	-0.707***	-0.461*	-0.731***
	(0.249)	(0.247)	(0.255)	(0.256)	(0.266)	(0.261)	(0.273)
Log-likelihood	-728.376	-710.130	-764.693	-670.573	-728.344	-706.327	-666.164
Newton-Raphson maximization	17	17	17	17	17	17	17
Free parameter	16	16	16	17	17	17	18

* p<0.05

** p<0.01

*** p<0.001

According to the results based on our control variables, the *Gender* variable heavily affects new link formation, where a female-female pair is less likely to become friends than male-female and male-male pairs. There was no serious effect based on *Language*, as the results only showed a higher likelihood for Korean-bilingual pairs making friends.

### Panel linear regression analysis (DV: Offline interaction)

To understand the process of a prior online network influencing offline behavior, which in turn affects the final network formation, a panel linear regression with the dependent variable as the log scale *Interaction* was performed (Table 4). As our data was gathered through 7 different social events at different time periods, we used *Party ID* dummy variables to produce a time-fixed model. There were 3 regression models: Model 1 and 2, which incorporate one of our two independent variables, online *Degree* of a pair and *Mutual Friends* at the social event, and Model 3, which incorporates both independent variables. Model 1 tests for the direct relationship between the online *Degree* of a pair and their offline *Interaction*. There was a slight negative and significant relationship between the amount of *Interaction* and the *Degree* of the observed pair at -0.0123 (p<0.01), indicating that the number of connections that a pair has is negatively associated with the amount of interaction between the pair. Model 2 tests for the relationship between the presence of *Mutual Friends* at the social event and offline *Interaction*, which showed no significant relationship between the presence of mutual friends and interaction. Model 3 analyzes both online *Degree* and the presence of *Mutual Friends* in relation to offline *Interaction* between the pair of participants. The *Degree* variable retains its negative significance at -0.0130 with p<0.01, while *Mutual Friends* at the social event is nonsignificant, as in Model 1. Factors from the prior social network structure affecting offline behavior were selective. The adjusted R^2^ value of the Model 3 is the highest, which indicates that Model 3 is the best-fitting model.

**Table 4:** Panel linear regression (DV: log(Interaction+1)).

Variables	Model 1	Model 2	Model 3
Intercept	3.590***	3.477***	3.582***
(s.e.)	(0.0926)	(0.0890)	(0.0934)
**Independent variables**			
Pair degree	-0.0123***		-0.0130***
	(0.00343)		(0.00359)
Mutual Friend (binary)		-0.0178	0.0326
		(0.0450)	(0.0471)
**Control Variables**			
Already friends (binary)	0.369***	0.291***	0.359***
	(0.0527)	(0.0514)	(0.0546)
Gender MF	0.466***	0.449***	0.4654***
	(0.0638)	(0.0638)	(0.0638)
Gender MM	0.747***	0.719***	0.747***
	(0.0675)	(0.0671)	(0.0675)
Language IE	-0.225***	-0.240***	-0.226***
	(0.0683)	(0.0683)	(0.0683)
Language II	-0.395*	-0.411**	-0.398*
	(0.158)	(0.158)	(0.158)
Language KE	-0.454***	-0.443***	-0.455***
	(0.0533)	(0.0533)	(0.0533)
Language KI	-0.428***	-0.424***	-0.428***
	(0.0709)	(0.0710)	(0.0709)
Language KK	-0.558***	-0.509***	-0.560***
	(0.0664)	(0.0650)	(0.0665)
Party ID 2	0.406***	0.374***	0.412***
	(0.0670)	(0.0669)	(0.0676)
Party ID 3	0.665***	0.692***	0.668***
	(0.0781)	(0.0780)	(0.0782)
Party ID 4	0.512***	0.467***	0.520***
	(0.0660)	(0.0655)	(0.0669)
Party ID 5	0.0724	0.0645	0.0838
	(0.0669)	(0.0688)	(0.0689)
Party ID 6	0.304***	0.326***	0.318***
	(0.0732)	(0.0758)	(0.0757)
Party ID 7	0.453***	0.465***	0.468***
	(0.0713)	(0.0747)	(0.0745)
Adj. R^2^	0.122	0.119	0.122
Cases N	3703	3703	3703
Individuals n	2937	2937	2937
Periods T	1–7	1–7	1–7

* p<0.05

** p<0.01

*** p<0.001

One of the studied variables, *Already Friend*, shows that interaction occurs more between pairs who are already friends on Facebook, with positive significance at 0.359 (p<0.01). Participants who are already friends tend to interact more with each other with those who are not.

The control variables, *Gender* and *Language*, showed significant effects throughout the analysis. The *Gender* dummy variable showed that female-female pairs had much less interaction, while male-female and male-male pairs had greater interaction. The *Language* variable showed cultural differences between international and Korean participants, as English-English speaking participant (EE) pairs showed the most interaction, while Korean-Korean pairs (KK) showed the least, possibly because of cultural reasons. The results for fluent bilingual participants (ethnically Korean) show that they interacted with English-speaking participants as did those in English-English pairs, showing relatively high interaction, but interacted less with Korean and other bilingual participants (KI and II). In general, the *Language* variable did not reveal less interaction due to language barriers, as we predicted; rather, it showed social behavior differences between Korean and non-Korean cultures.

## Discussion

The main goal of this study was to articulate the effects of online predictors with offline interaction for social tie formation. According to the outcomes of our study, social predictors from existing online networks can be used to predict network evolution through the conventional preferential attachment model, which relies on the degree centralities of network participants [19]. This finding indicates that the degree centrality of a pair contains social resources from the existing network that drive the network formation and are not accounted for by offline interactions at social events.

However, the common neighbor effect, which was believed to promote social connections by Newman [8], actually showed opposite tendencies in the formation of new social connections (Table 3). Here, the presence of mutual friends may be a factor that brings two participants together, but under the circumstances of offline social events, this effect is nullified because it is the social event that brings participants together, not mutual friends. The common neighbor effect may only be visible in natural friendship over a longitudinal observation period in which mutual friends introduce friends to each other rather than through pre-designed social events that eliminate the necessity of mutual friends to bring two unconnected people together.

Furthermore, the presence of mutual friends has negative effects on making new social ties. This result may be due to the unique triangular network structure formed by two interacting participants who are not yet connected, joined by their shared mutual friend. The *Already Friends* variable in the regression with offline *Interaction* as the dependent variable (Table 4) promotes interaction between friends. If a mutual friend is present between a pair, then each node of the pair interacts more with the common mutual friend than with one another. As participants are already friends with the common mutual friend and tend to interact more with the common mutual friends than with each other, this leads to relatively less interaction between the observed pair given the limited duration of the social events. The presence of mutual friends may be a distraction to the pair when interacting with each other.

As our study verifies the predicative power of conventional network prediction models when offline interactions are quantified, we provide balanced grounds for evaluating the qualities of online and offline networks by observing both throughout the process of social network formation. The addition of real human behaviors to the network prediction model leads to the dual online and offline social network model that conceptualizes the formation of social ties via existing online networks and offline social events. Therefore, our newly established dual-dimensional model can be applied to components of intra/inter-organizational social networks as well as non-social networks with online and offline layers.

Moreover, our research framework is unique in that we designed social experiments in the form of offline networking events to observe network evolution firsthand. Utilizing our interaction measurement method, we have quantified the actual offline interaction between individuals. While conventional models predict growth of a network according to characteristics of the network itself, our framework allows two-dimensional analysis from both online and offline perspectives, as aforementioned. We expect future social network studies to utilize our distinct framework to understand the causal effect of catalytic factors in network evolutions.

Our study establishes the initial step towards the systematic representation of online and offline social factors on an integrated basis. Today, many social networking platforms are seeking ways to facilitate offline interaction among users. Many platforms already utilize geolocation and demographic data from users’ mobile devices to assist in offline meetups with nearby friends and with people with similar interests in real time. These online platform services can design new services to integrate online and offline domains ubiquitously based on our understanding of the social network formation process.

Although we quantified social network formation from existing online networks within the bounds of all event participants to understand offline interactions, our data was limited to event participants only, due to Facebook privacy issues. To obtain the most accurate data from existing social networks, it would be best to observe not only the network of our event participants but also the entire networks to which they belong. Future research could design closed on-offline network settings to monitor the formation of networks from the beginning and control unseen effects of outside networks. As explained above, our research participants were not in a closed network. They were free to interact at their will outside of social event settings. Our research accounted only for interactions measured through the social events and could not control for possible extra interactions outside of the measured boundaries. We expect further studies to combine network analysis techniques with our multi-dimensional model to comprehend a wider range of social contexts in diverse dimensions. These research results will augment the human ability to manage on-offline social networks, enhancing social and business strategies and eventually sociological and political policies.
